# The association of post-conization pregnancy with subsequent cervical lesions: evidence from a nationwide cohort in Sweden

**DOI:** 10.1186/s12916-025-04541-w

**Published:** 2025-12-29

**Authors:** Huan Yi, Jimiao Huang, Naiqi Zhang, Jan Sundquist, Kristina Sundquist, Xiangqin Zheng, Jianguang Ji

**Affiliations:** 1 College of Clinical Medicine for Obstetrics & Gynecology and Pediatrics, Fujian Medical University, Fuzhou, Fujian 350001 China; 2 Center for Primary Health Care Research, Department of Clinical Sciences Malmö, Lund University, Lund, Sweden; 3 Fujian Province Key Clinical Specialty for Gynecology, Fujian Maternity and Child Health Hospital, Fujian Key Laboratory of Women and Children’s Critical Diseases Research, National Key Gynecology Clinical Specialty Construction Institution of China, Fuzhou, China; 4 Center for Community-based Healthcare Research and Education (CoHRE), Department of Functional Pathology, School of Medicine, Shimane University, Matsue, Japan; 5 University Clinic Primary Care Skåne, Malmö, Region Skåne Sweden; 6 Faculty of Health Sciences, University of Macau, Taipa, Macau SAR China; 7 Cancer Center, Faculty of Health Sciences, University of Macau, Taipa, Macau SAR China

**Keywords:** Cervical intraepithelial neoplasia (CIN), Conization, Delivery, Pregnancy

## Abstract

**Background:**

Women who have undergone cervical conization may still experience subsequent pregnancies and delivery. However, it remains unknown whether pregnancy, associated with immune tolerance, might increase the risk of subsequent cervical lesions. This study aims to address this knowledge gap by utilizing the nationwide Swedish registers.

**Methods:**

A total of 60,895 women diagnosed with cervical intraepithelial neoplasia in Sweden between January 1997 and December 2017 and treated with conization were identified through the Swedish National Patient Register and followed for subsequent cervical lesions. Time-dependent Cox regression was used to examine the association of post-conization pregnancies with subsequent cervical lesions.

**Results:**

Among the 60,895 women who underwent conization in Sweden, 15,200 (25%) had post-conization pregnancies and showed a higher incidence of subsequent cervical lesions (hazard ratio = 1.32, 95% confidence interval = 1.13–1.53) compared to women without pregnancies. The increased risk of subsequent cervical lesions was observed only in women who had a pregnancy within 3 years after conization (adjusted hazard ratio = 1.39, 95% confidence interval = 1.19–1.63).

**Conclusions:**

Our study demonstrates that post-conization pregnancies are associated with a higher risk of subsequent cervical lesions compared to women without pregnancies. The risk was particularly significant in women who had a pregnancy within 3 years after conization, suggesting that those who become pregnant within 3 years after conization will need close clinical monitoring.

**Supplementary Information:**

The online version contains supplementary material available at 10.1186/s12916-025-04541-w.

## Background

The prevalence of human papillomavirus (HPV) is highest among women under 35 years of age across various regions of the world, which coincides with the peak childbearing age group [1]. Persistent high-risk HPV infection increases the risk of cervical lesions progressing to cervical cancer [2]. Cervical conization is a common treatment for cervical intraepithelial neoplasia and is effective in preventing the development of cervical cancer [3]. However, women of childbearing age may still have reproductive needs after conization. It is known that pregnancy induces immune tolerance [4]; thus, post-conization pregnancy may impair HPV clearance and increase the risk of subsequent cervical lesions. Unfortunately, it remains unclear whether pregnancy, with its associated immune tolerance, influences the recurrence or progression of cervical lesions, an issue that warrants further investigation.

In this nationwide cohort study in Sweden, we aimed to investigate the association of post-conization pregnancies with subsequent cervical lesions. By integrating data from multiple nationwide registers in Sweden, we evaluated the risk of subsequent cervical lesions among 15,200 women who had post-conization pregnancies and explored whether the timing of pregnancy after conization influenced the risk of subsequent cervical lesions.

## Methods

### Data sources

The study cohort was approved on 6 February 2013 by the Lund Regional Ethical Review Board (Dnr 2012/795 and later amendments).

We identified all Swedish women aged 40 or younger who were diagnosed with cervical intraepithelial neoplasia (CIN) 2/3 (International Classification of Disease (ICD)−10 code: N87) or carcinoma in situ of the cervix uteri (ICD-10 code: D06) and who underwent conization between January 1997 and December 2017 from the Swedish National Patient Register. This register included the date of hospitalization, clinical diagnosis, and surgical procedures. Women with a follow-up time of less than 1 year were excluded from the study to ensure that all participants had sufficient time to potentially become pregnant following conization. Pseudonymized numbers were assigned to individuals to ensure linkage among Swedish nationwide registers while protecting the privacy and integrity of the data. This study was conducted following the ethical standards set forth in the Declaration of Helsinki [5]. The study methodology was in accordance with the Strengthening the Reporting of Observational Studies in Epidemiology (STROBE) [6] and the REporting of studies Conducted using Observational Routinely collected health Data (RECORD) [7] reporting guidelines for cohort studies.

### Assessment of exposures

Women who became pregnant following conization were identified using the Swedish Medical Birth Register. This register was started in 1973, which provides comprehensive data on all pregnancies resulting in childbirth in Sweden. Post-conization pregnancy was defined as women who became pregnant after conization and had a successful delivery.

### Assessment of outcomes

#### Subsequent cervical lesions

We linked all the women with conization to the National Patient Register and the Swedish Cancer Registry to identify the subsequent cervical lesions by using ICD code of D06 for carcinoma in situ or C53 for cervical cancer.

The follow-up was started on the date of conization and ended on the date of diagnosis of subsequent cervical lesions, death from any cause, or the end of the study period (December 31, 2018), whichever came first. To minimize bias from insufficient observation time, we excluded women with follow-up time shorter than 12 months.

### Assessment of covariates

Potential confounding factors were obtained from the National Patient Register, Statistics Sweden’s Total Population Register, and Population Housing Census, which included birth year, year of conization, education (1–9, 10–11, and > = 12 years) [8], income (lowest, middle-low, middle-high, highest), region (big cities, southern Sweden, northern Sweden, missing), age at first delivery (never, < = 25 years, 26–30 years, 31–35 years, 36–40 years), parity before the conization (0, 1, 2, 3, > = 4), obesity (yes/no), history of hormonal therapy (yes/no), and CCI (0, 1, 2, > = 3). Recognizing that age at first childbirth may serve as a surrogate for the duration of sexual activity and potential HPV exposure, we included age at first childbirth to account for its potential confounding risk. As comorbidity is an important factor affecting health conditions, we calculated the CCI based on a total of 17 categories [9].

### Statistical analysis

Continuous covariates were expressed as mean ± standard deviation, while categorical covariates were presented as counts and percentages. We employed a proportional Cox regression model to determine the hazard ratio (HR) and 95% confidence interval (CI) of subsequent cervical lesions. The regression analysis was adjusted for clinical and demographic factors (Table 1). Post-conization pregnancy was treated as a time-dependent variable, with women initially categorized as unexposed until they were pregnant. We stratified the results according to the time intervals between conization and post-conization pregnancy to determine a safe timeframe during which women can undergo childbirth without an increased risk of subsequent cervical lesions. Additionally, we stratified the patients by the year of conization (1997–2006 and 2007–2017).

**Table 1:** Baseline demographic and clinical characteristics between pregnant women and nonpregnant women with conization

Characteristics	Pregnant women (*n* = 15,200)		Nonpregnant women (*n* = 45,695)	
Age	Number	%	Number	%
< = 25	3946	26.0	8737	19.1
26–30	6779	44.6	13,765	30.1
31–35	3604	23.7	12,144	26.6
36–40	871	5.7	11,049	24.2
Year of conization				
1997–2001	4270	28.1	4906	10.7
2002–2006	5864	38.6	6122	13.4
2007–2011	4598	30.3	9995	21.9
2012–2017	468	3.1	24,672	54.0
Education				
0–9	923	6.1	4396	9.6
10–11	6328	41.6	22,287	48.8
> = 12	7949	52.3	19,012	41.6
Income				
Low	2259	14.9	12,974	28.4
Medium–low	3347	22.0	11,875	26.0
Medium–high	4203	27.7	11,025	24.1
High	5391	35.5	9821	21.5
Region				
Big cities	2442	16.1	9426	20.6
Southern Sweden	10,414	68.5	29,395	64.3
Northern Sweden	2344	15.4	6872	15.0
Missing	0	0.0	2	0.0
Diagnosis				
Carcinoma in situ	8908	58.6	29,756	65.1
CIN2/3	6292	41.4	15,939	34.9
CCI				
0	14,348	94.4	42,077	92.1
1	775	5.1	3136	6.9
2	58	0.4	325	0.7
> = 3	19	0.1	157	0.3
Age at first childbirth				
Never	9836	64.7	23,213	50.8
< = 25	2966	19.5	13,130	28.7
26–30	1683	11.1	6909	15.1
31–35	651	4.3	2234	4.9
36–40	64	0.4	209	0.5
Parity before conization				
0	9836	64.7	23,213	50.8
1	3288	21.6	7145	15.6
2	1596	10.5	11,064	24.2
3	363	2.4	3374	7.4
> = 4	117	0.8	899	2.0
COPD				
No	14,599	96.0	43,318	94.8
Yes	601	4.0	2377	5.2
Obesity				
No	15,131	99.5	44,897	98.3
Yes	69	0.5	798	1.7
History of hormonal therapy				
No	15,151	99.7	45,369	99.3
Yes	49	0.3	326	0.7

*CCI *Charlson comorbidity index, *COPD *chronic obstructive pulmonary disease, *CIN *cervical intraepithelial neoplasia

We conducted a sensitivity analysis to further validate our hypothesis regarding the potential impact of immune tolerance during pregnancy on subsequent cervical lesions. Specifically, we examined the association between abortion and susceptibility to subsequent cervical lesions, considering that the immune tolerance period in women who experienced abortion might be shorter compared to those with full-term pregnancies, potentially having less effect on the subsequent risk of cervical lesions. All analyses were conducted using SAS, version 9.4 (SAS Institute, Cary, NC, USA) and R statistical package, version 4.3.1 and SPSS version 26.0 (IBM Corp., USA).

## Results

**Fig. 1:**
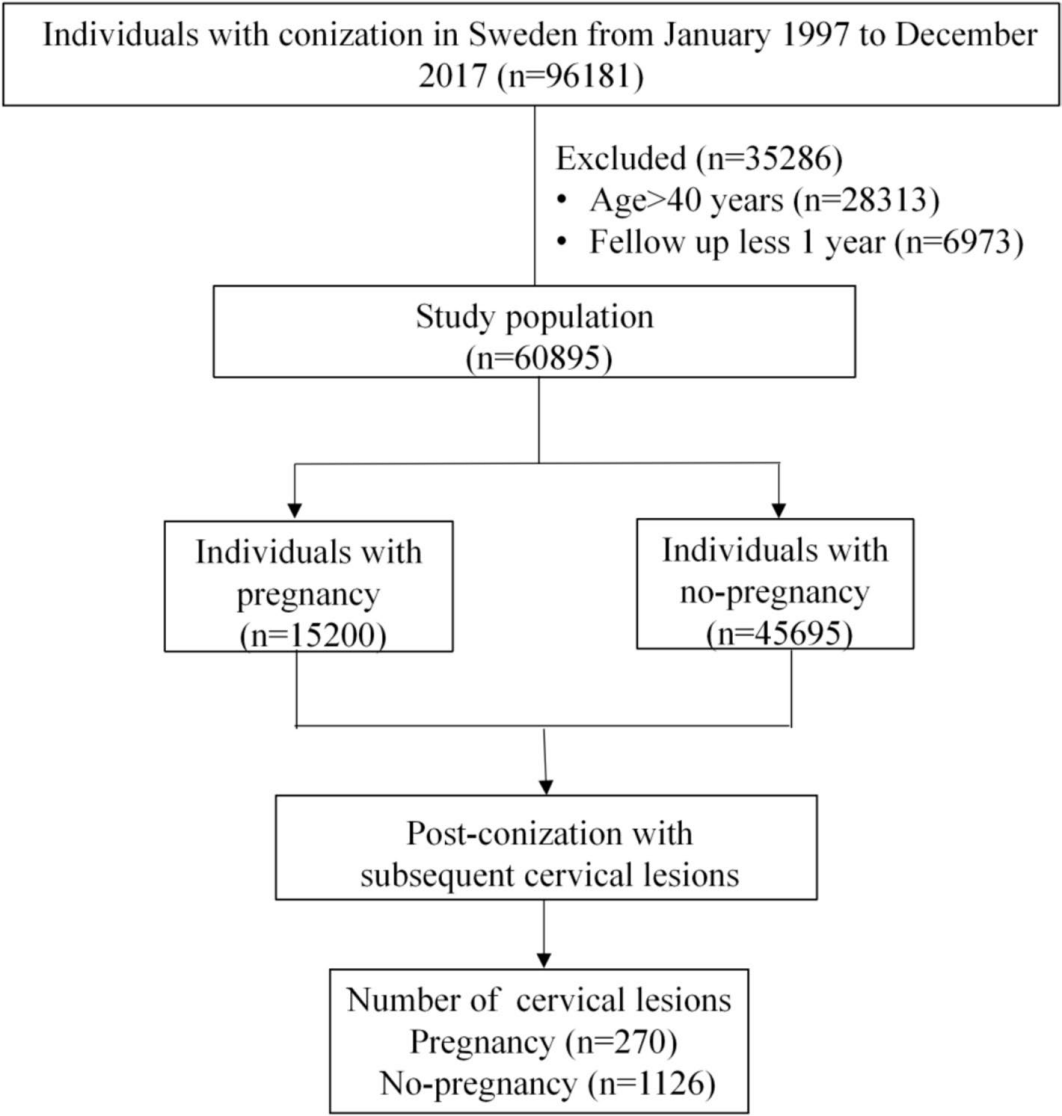
Flow chart of participants involved in this study

A total of 60,895 women underwent conization between 1997 and 2017 in Sweden, and 25% of them had post-conization pregnancies, as shown in Fig. 1. All the characteristics listed in Table 1 were adjusted for in the final multivariable regression model.

Post-conization pregnancy was associated with an increased risk of subsequent cervical lesions, with an adjusted HR of 1.32 (95% *CI* = 1.13–1.53). The stratified analysis found that pregnancy was linked to the occurrence of carcinoma in situ of cervix uteri, with an adjusted HR of 1.36 (95% *CI* = 1.16–1.59), whereas it had no significant effect on cervical cancer. In the analysis stratified by year of conization, the association was largely consistent, with an adjusted HR of 1.26 (95% *CI* = 1.02–1.56) for women who received conization between 1997 and 2006 and 1.31 (95% *CI* = 1.05–1.62) for women who received conization between 2007 and 2017 (Table 2). Among the 23 cases that progressed to cervical cancer, the clinical stages were distributed as follows: IA1 (5 cases), IA2 (2 cases), IB1 (6 cases), IIA (1 case), IIB (1 case), IVB (1 case), and unknown (7 cases). In the pregnancy group (*n* = 15,200), 688 women (4.53%) underwent repeat excisional treatment after pregnancy. Of the 270 women in this group who experienced a subsequent cervical lesion, 107 (39.63%) received a repeat excision.

**Table 2:** Hazard ratios and 95% confidence intervals for the risk of subsequent cervical lesions associated with post-conization pregnancy

	No. of individuals	Follow-up years	No. of outcome	IR^a^	Crude			Adjusted^b^		
					HR	95% CI	*p*-value	HR	95% CI	*p*-value
Overall										
Non-pregnancy	45,695	356,019	1126	3.16	1			1		
Pregnancy	15,200	211,091	270	1.28	1.19	1.03–1.37	0.018	1.32	1.13–1.53	< 0.001
Type of lesions										
CIN	15,200	211,104	254	1.20	1.24	1.07–1.43	0.005	1.36	1.16–1.59	< 0.001
Cervical cancer	15,200	212,771	23	0.11	0.84	0.51–1.38	0.485	1.28	0.76–2.18	0.353
Time interval between conization and pregnancy										
< 3 years	8111	102,328	209	2.04	1.28	1.10–1.49	0.002	1.39	1.19–1.63	< 0.001
> = 3 years	7089	108,763	61	0.56	0.89	0.67–1.18	0.405	0.98	0.73–1.31	0.873
Year of conization										
1997–2006	10,134	163,559	156	0.95	1.16	0.95–1.42	0.155	1.26	1.02–1.56	0.033
2007–2017	5066	47,532	114	2.40	1.34	1.09–1.65	0.005	1.31	1.05–1.62	0.015
Sensitivity analysis										
Abortion	2370	19,480	33	1.69	1.35	0.95–1.91	0.093	1.19	0.84–1.69	0.318

*IR *incidence rate, *HR *hazard ratio, *CI *confidence intervals, *CCI *Charlson comorbidity index, *COPD *chronic obstructive pulmonary disease, *CIN *cervical intraepithelial neoplasia

^a^*IR*, incidence rate, per 1000 person-year

^b^Adjusted for age, year of conization, education, region, income, diagnosis, COPD, age at first childbirth, parity before conization, obesity, history of hormonal therapy, and CCI

Additionally, we found that pregnancy within 3 years after conization had a significant effect on subsequent cervical lesions (adjusted *HR* = 1.39, 95% *CI* = 1.19–1.63), whereas women with pregnancy 3 years after conization were not associated with subsequent cervical lesions. For the sensitivity analyses, we found that in women with abortion after conization, there was no statistically significant effect on subsequent cervical lesions, with an adjusted HR of 1.19 (95% *CI* = 0.84–1.69).

## Discussion

This population-based nationwide cohort study is, to the best of our knowledge, the first to investigate whether post-conization pregnancy affects subsequent cervical lesions in women treated with conization for carcinoma in situ or CIN 2/3. Our findings indicate that post-conization pregnancy is associated with an increased risk of subsequent cervical lesions, possibly due to the immune-tolerant state during pregnancy, which may impair the body’s ability to clear HPV. Interestingly, the increased risk of subsequent cervical lesions was observed only in women who became pregnant within 3 years of conization, suggesting that delaying pregnancy for at least 3 years might be a reasonable recommendation for women planning to conceive.

Persistent high-risk human papillomavirus (hr-HPV) and subsequent hr-HPV reinfection are key determinants of cervical lesion [10, 11]. Studies have shown that gestational HPV infection can evade detection by the immune system, allowing it to replicate without interference, which aligns with the hypothesis that the typical hormonal and immunological tolerance during pregnancy could impair the immune system’s ability to clear HPV infection or potentially trigger viral activation [12–14]. Therefore, latent HPV is prone to be activated again during pregnancy, leading to the recurrence of HPV infection and cervical lesion [15]. Previous studies have also shown that the increased levels of estrogen and growth hormone during pregnancy can activate the expression of the HPV gene, thus affecting the natural regression of HPV [16, 17]. Both in vitro and in vivo experimental and epidemiological studies have reported that estrogen is a risk factor for HPV activation [18–22]. Compared with short-term estrogen exposure, long-term (more than 5 years) oral hormonal contraception (containing estrogen) is a notable risk factor for adverse outcomes of HPV infection and cervical lesions [23]. However, we were unable to assess the impact of hormonal therapy on HPV persistence and cervical lesions due to the lack of relevant data; thus, we could not draw a concrete conclusion based on our analyses.

Previous studies have revealed that during pregnancy, the persistence rate of CIN 2/3 lesions is substantial, ranging between 50.0% and 59.0%, with an additional 1% to 2.9% of these lesions demonstrating progression to more severe stages [24, 25]. Nevertheless, studies with small sample sizes showed that CIN persistence and progression rates were lower in pregnant women than in nonpregnant women [26]. It is worth noting that all the previous studies had shorter follow-up times, which might lead to underdiagnosis of cervical lesions [27]. In this study, women with conization have been followed for up to 21 years, which guarantees sufficient follow-up time to identify subsequent cervical lesions. Additionally, our study explored the risk of subsequent cervical lesions among women with abortion after conization. The results suggest that abortion has a nonsignificant influence on the subsequent cervical lesions, but the overall trend suggests that abortion might also have a contributory effect on the pathology of CIN.

With the widespread use of cytology and colposcopy, coupled with trends such as late marriage and delayed childbearing, an increasing number of unmarried and childless women are being diagnosed with CIN 2/3 or carcinoma in situ of the cervix. Many of these women undergo cervical conization and later go on to experience pregnancy and childbirth [28, 29]. Our study indicates that post-conization pregnancies, especially those within 3 years, significantly elevate the risk of subsequent cervical lesions. This insight is crucial for guiding clinical advice on the optimal timing for pregnancy after conization. However, the reasons for the higher risk of subsequent cervical lesions within 3 years remain to be explored in further studies. Some studies suggest that conization may disrupt the local microenvironment of the cervix, thereby increasing the risk of cervical infections [30]. Furthermore, sexual activity during the post-conization healing period may elevate the risk of HPV infection [31].

Studies have shown that adaptive physiological changes occur during pregnancy [32]. The progression or recurrence of cervical lesions may be linked to immunological tolerance during pregnancy [33]. Our population-based cohort study supports this hypothesis, suggesting that pregnancy-induced immune tolerance impairs the host’s ability to clear HPV and increases susceptibility to infection. However, the dynamics of subsequent cervical lesions during pregnancy and postpartum remain poorly understood, and the underlying mechanisms driving the long-term effects of pregnancy on cervical lesion progression or recurrence are still unexplored.

To our knowledge, the current study was the first study to estimate the relationship of post-conization pregnancy with cervical lesions. The major strength of the current study was that the nationwide register in Sweden can effectively minimize selection bias and avoid recall bias. Additionally, the larger sample sizes and the longer follow-up time guarantee statistical power and prevent reversal causality. One limitation of the current study is that we were unable to determine whether the subsequent lesions after conization were the result of the initial lesion or new lesions from separate high-risk HPV infections due to the absence of HPV examination and vaccination data. Additionally, some potential confounders are lacking in our study, such as lifestyle factors, including smoking, alcohol consumption, and dietary habits, which might affect the risk of subsequent cervical lesions. While we have adjusted for COPD as a proxy for smoking and education in regression models, which are highly correlated with lifestyle factors, this may only partially mitigate their confounding effects. We acknowledge that residual confounding may still exist and could influence our findings.

## Conclusions

In summary, this population-based cohort study suggests that women who are pregnant after conization have a higher risk of subsequent cervical lesions compared to nonpregnant women. Hence, regular cervical examination is required among women with post-conization pregnancy, especially for women who are pregnant within 3 years after the conization.

## Supplementary Information

Supplementary Material 1.

## Data Availability

The data based on the Swedish register are not publicly available due to Swedish law and protecting patients’ privacy, and the combined set of data used for the analysis presented in this study can only be made available from the appropriate Swedish authorities (the Swedish National Board of Health and Welfare (https://www.socialstyrelsen.se/en) and Statistics Sweden (https://www.scb.se/en)), for researchers who meet the criteria for access to confidential data. The data used for this study and additional information are available from the corresponding author on reasonable request.

## Abbreviations

HPV: Human papillomavirus
hr-HPV: High-risk human papillomavirus
CIN: Cervical intraepithelial neoplasia
ICD: International Classification of Disease
IR: Incidence rate
HR: Hazard ratio
CI: Confidence interval
CCI: Charlson comorbidity index
COPD: Chronic obstructive pulmonary disease
STROBE: Strengthening the Reporting of Observational Studies in Epidemiology
RECORD: REporting of studies Conducted using Observational Routinely Collected health Data
